# Oxidative Stress and Antioxidative Activity in Leaves and Roots of Carrot Plants Induced by *Candidatus* Phytoplasma Solani

**DOI:** 10.3390/plants10020337

**Published:** 2021-02-10

**Authors:** Petar Mitrovic, Ivica Djalovic, Biljana Kiprovski, Sonja Veljović Jovanović, Vojislav Trkulja, Aleksandra Jelušić, Tatjana Popović

**Affiliations:** 1 Institute of Field and Vegetable Crops, National Institute of the Republic of Serbia, Maxim Gorki 30, 21000 Novi Sad, Serbia; petar.mitrovic@ifvcns.ns.ac.rs (P.M.); biljana.kiprovski@ifvcns.ns.ac.rs (B.K.); 2 Institute for Multidisciplinary Research, University of Belgrade, 11030 Belgrade, Serbia; sonjavel@imsi.bg.ac.rs (S.V.J.); aleksandra.jelusic@imsi.bg.ac.rs (A.J.); 3 Department of Plant Protection, Agricultural Institute of Republic of Srpska, Knjaza Milosa 17, 78 000 Banja Luka, Bosnia and Herzegovina; vtrkulja@blic.net; 4 Institute for Plant Protection and Environment, Teodora Drajzera 9, 11040 Belgrade, Serbia; tanjaizbis@gmail.com

**Keywords:** *Daucus carota* L., phytoplasma, antioxidants, pigments, polyphenols, sugar

## Abstract

The present study examined the effects of *Candidatus* Phytoplasma solani infection on antioxidative metabolism in leaves and roots of carrot (*Daucus carota* L.). Disease symptoms appeared at the end of June in the form of the chlorosis on some of the leaves, which became intensely red one week later, while the previously healthy leaves from the same branch becme chlorotic. A few days later, all leaves from the infected leaf branch were intensely red. Infected plants also had slower growth compared to the healthy ones with fewer leaf branches developed. The roots of infected plants were less developed, seared, or gummy with or without brown-colored root hair. The presence of the pathogen was detected by sequencing the 16S rRNA. National Center for Biotechnology Information (NCBI) BLAST analyses of the obtained sequence revealed 100% identity of tested strain with deposited Ca. Phytoplasma solani strains from various countries and hosts, all belonging to the “stolbur” group (16SrXII-A). Identity of 99.74% was found when the tested Serbian strain (MF503627) was compared with the reference stolbur strain STOL11 (AF248959). The oxidative damage of membranes in carrot cells was accompanied by a decrease in the content of photosynthetic pigments. Furthermore, for the determination of specific scavenging properties of the extracts, in vitro antioxidant assay was performed. In phytoplasma-infected carrot leaves, there was a greater reduction in the level of glutathione content (GSH); however; flavonoids and anthocyanidins seem to be responsible for the accompanied increased antioxidative capacity against hydroxyl radical and hydrogen peroxide.

## 1. Introduction

Phytoplasmas are plant pathogens from the class Mollicutes that inhabit phloem sieve elements of host plants [1]. A wide range of visible symptoms caused by phytoplasmas are described: leaves yellowing; phyllody; virescence; Witches’ broom or proliferation; heavy leaves with thick laminas, edges rolled up or down, stiff to the touch and brittle; small malformed crinkled leaves; thick bark above the phloem interruption point; phloem necrosis or vein necrosis; vascular discoloration; leaf veins that are pale or purple, prominent, and winding; leaf petioles that are shorter and thicker than regular leaves; small round fruits with long petioles; basal suckers, even visible from a distance; rosetting occurring in shoot apices [2,3]. Other symptoms are more generic (for groups of pathogens that are not phytoplasmas) such as chlorosis, necrosis, flower abortion, small fruits, stunting, decline, etc. [2]. Symptoms such as chlorophyll photooxidation are the result of oxidative stress, which occurs due to the accumulation of reactive oxygen species (ROS), which is a common feature of most abiotic and biotic stresses of plants. On the other hand, an “oxidative burst”, a phenomenon related to the pathogen attack, initiates a cascade of signaling pathways, including the plant stress hormone network. Plant hormones such as jasmonic (JA) and salicylic acid (SA) participate directly or indirectly in the generation of the oxidative burst, superoxide, and hydrogen peroxide in the extracellular apoplast–cell wall matrix by enzymes such as the plasmalemma-localized NADPH oxidases and peroxidases [4]. 

Members of the *Candidatus* Phytoplasma genus are obligate parasites that require plant host and insect vectors for spread and survival. Unlike those biotrophic pathogens that do not require insect vectors, and which activate the JA-dependent pathway but inhibit the SA-dependent pathway [5], phytoplasma appears to have a contrasting effect on the plant defense system. It has been suggested that some of the secreting effectors, which are systematically transported through phloem sieve cells, suppress JA synthesis, and thus compromise the plant defense system [6]. Based on the data on the regulation of phenylalanine ammonia-lyase activity (PAL) accompanied by an accumulation of hydroxycinnamates and inhibition of flavonoid biosynthesis in phytoplasma-infected plants, Musetti [7] suggested that polyphenols are involved in plant defense against phytoplasma. In our previous study, similar changes in the pattern of inducible polyphenols in *Oenothera biennis* plants were obtained when infected by *Candidatus* Phytoplasma solani [8]. SA plays a crucial role in numerous plant defense responses, such as local and systemic pathogen-induced defense gene activation, the oxidative burst, and pathogen-induced cell death [9,10,11,12,13]. In addition to their role in signaling (SA), some classes of polyphenols (hydroxycinnamates and flavonoids) are considered important antioxidants [14,15].

In Serbia, phytoplasmas have been found in cereals, vegetables, spices, and medicinal plants [16,17,18,19]. Since the beginning of the XXI century, the carrot (*Daucus carota* L.) has been considered an important vegetable crop in Serbia, showing a trend of increasing the surfaces on which it is grown over the years. The first occurrence of phytoplasmas on carrot in Serbia has been reported by Duduk et al. [20]. In later work, Duduk et al. [21] stated that the aster yellows phytoplasma belonging to subgroup 16SrI-A was prevalent in Serbian carrot fields. In the last few years, the symptoms of reddening or purpling leaves, as well as smaller and poor main roots have been noticed in several production regions of Serbia. 

The aim of the study was to identify the strain of Ca. Phytoplasma solani and to test the response to the infection in diseased carrot plants. The extent of oxidative stress induced by the infection was measured by levels of reduced glutathione content (GSH) and polyphenols. Damage in phytoplasma-infected carrot plants was determined by measuring a malondialdehyde (MDA), which is a product of oxidative membrane degradation, and bleaching of photosynthetic pigments. Furthermore, in vitro assay for the determination of specific scavenging properties of leaves and roots extracts was performed to reveal the ability of tested plants to cope with oxidative burst caused by determined phytoplasma pathogen.

## 2. Results

### 2.1. Symptoms Observation 

**Figure 1:**
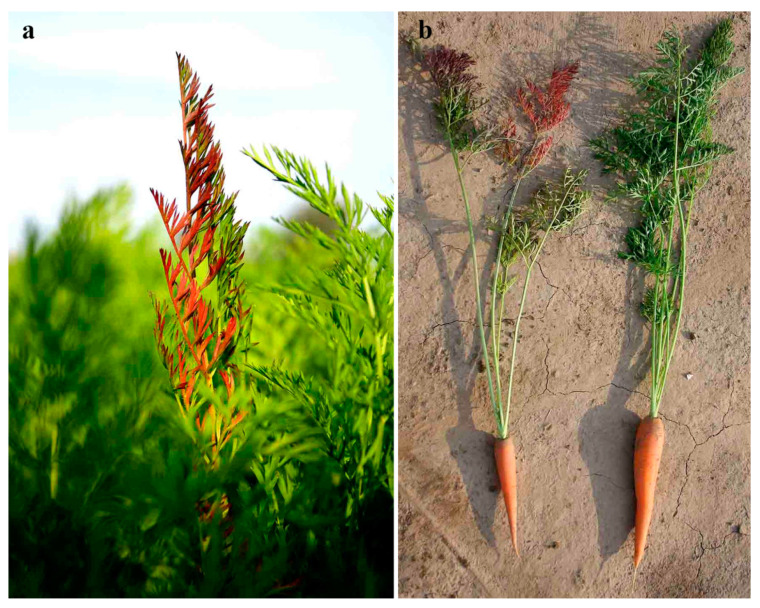
Symptoms of Ca. Phytoplasma solani on carrot plants (**a**) leaves with intensive red color (**b**) diseased plant (left) and healthy plant (right).

The first disease symptoms were observed at the end of June, in the form of chlorosis on some of the leaves. One week later, chlorotic leaves became an intense red color, while the previously healthy leaves from the same branch become chlorotic. A few days later, all leaves from the infected branch were intensely red (Figure 1a). Infected plants had also slower growth compared to the healthy ones with fewer leaf branches developed (Figure 1b). The roots of infected plants were less developed, seared, or gummy with root hair brown colored or without them (Figure 1b).

**Figure 2:**
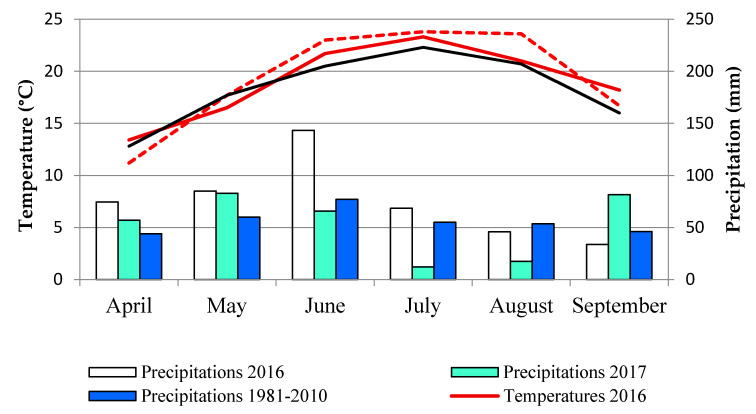
Meteorological data for study periods (2016–2017), location Futog, Vojvodina, Serbia.

During the colelection of carrot samples (end of June, beginning of July), climatic conditions (temperature, rainfall) did not contribute to the disease incidence, although they were different in both years. During June and July, average temeratures in 2016 (21.7 °C and 23.3 °C, respectively) were lower than in 2017 (23.0 °C and 23.8 °C, respectively) and precipitations in 2016 (143.2 mm and 68.4 mm, respectively) were higher than in 2017 (65.7 mm and 12.0 mm, respectively) (Figure 2). 

### 2.2. Molecular Identification of Candidatus Phytoplasma Solani

**Figure 3:**
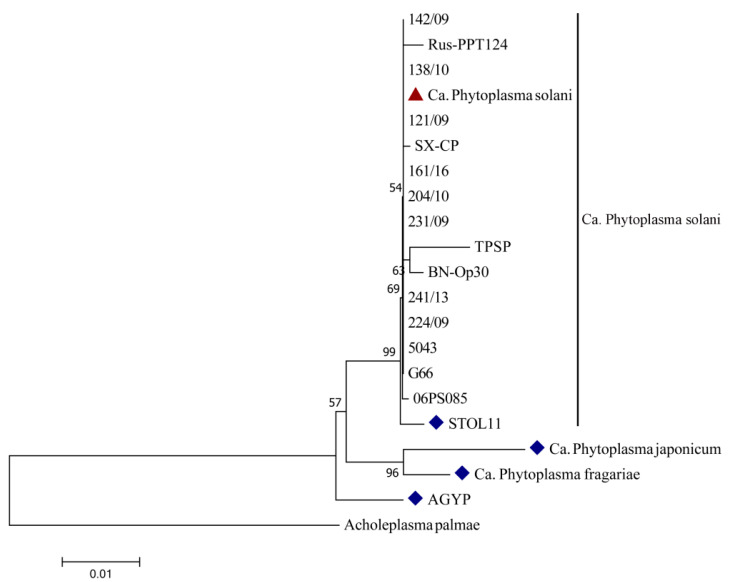
Neighbor-joining phylogenetic tree constructed with tested Serbian Ca. Phytoplasma solani strain from carrot and other Ca. Phytoplasma (solani, australiense, japonicum, and fragariae) strains from the NCBI database. Serbian Ca. The Phytoplasma solani strain from this study is marked with a triangle, and strains marked with a rhombus are reference strains for certain species.

One clear band on the position of 1784 bp was obtained after PCR amplification of tested Serbian strains from symptomatic carrot leaves using two universal primers pairs (P1/P7 and P1A/P7A). Band presence was not visible in any of the negative controls. National Center for Biotechnology Information (NCBI) BLAST analyses of the obtained sequences of Serbian carrot strains (1553 nt in size) revealed 100% identity with already deposited sequences of Ca. Phytoplasma solani strains from various countries and hosts (Table 1), all belonging to the Stolbur group (16SrXII). BLAST analysis also showed a 99.74% homology of Serbian carrot strains with reference stolbur strain STOL11 (AF248959). One Serbian strain was deposited to the NCBI database under the accession number MF503627. The constructed neighbor-joining phylogenetic tree, which is presented in Figure 3, shows relatedness among Serbian Ca. Phytoplasma solani carrot strain, reference, and other strains of this species, as well as reference Ca. Phytoplasma (australiense, japonicum, and fragariae) strains from the NCBI database. Despite differences in the host of origin, country of isolation, and symptoms they cause, all Ca. Phytoplasma solani strains were grouped in one phylogenetic tree cluster, showing no presence of genetic diversity among this species. Reference strains of Ca. Phytoplasma japonicum and Ca. Phytoplasma fragariae formed another group among the same cluster as Ca. Phytoplasma solani, showing greater phylogenetic relation among themselves than with Ca. Phytoplasma solani strains, including the Serbian strains from carrot Ca. The Phytoplasma australiense strain AGYP is separated and closely related to other tested species from the 16S rXII group, indicating that they share a common ancestor. 

### 2.3. Oxidative Stress and Antioxidative Activity

We observed that carrot plants at the same developmental stage responded differently to the infection with Ca. Phytoplasma solani in two successive years, based on a larger difference in lipid peroxidation between asymptomatic and symptomatic leaves and roots measured in 2017. However, in 2016, a smaller accumulation of sugars, reduced glutathione, and total polyphenols were accompanied by intensified cell membrane peroxidation in asymptomatic leaves and roots (Table 2). 

Almost no difference in the content of total sugars between symptomatic and asymptomatic plants was observed in 2016. However, a significant increase in total sugars was measured in symptomatic leaves in comparison to asymptomatic leaves of carrot grown in 2017. The increase in total sugars was accompanied by an almost threefold increase in GSH level in both symptomatic leaf and root. Despite the activation of antioxidative defense in plants with symptoms of infection, the degradation of membrane lipids was 10 times lower in intensity in plants collected in 2016, irrespective of the presence of symptoms. An impaired antioxidative response (GSH and antioxidative capacity) to infection with Ca. Phytoplasma solani in 2016 compared to 2017 resulted in about 50% and 20% higher oxidative stress (LP) in symptomatic leaves and roots, respectively. In both years, the activity of PAL was induced in leaves, but not in root after plant infection. A significant increase was observed only for 2017 (Table 3). 

A slight increase in the accumulation of polyphenols was observed in 2016 but not in 2017. Proanthocyanidins and anthocyanins had the opposite changes. While proanthocyanidins decreased in symptomatic plants, anthocyanins increased. The contents of both types of photosynthetic pigments, chlorophylls, and carotenoids decreased in plants with visible symptoms of infection. Contents of chlorophylls in leaves of symptomatic plants were significantly decreased in comparison to asymptomatic plants, contrary to anthocyanins, which accumulated in infected leaves (Table 4). 

Significant differences between tested years were observed for antioxidant response to pathogen infection when antioxidant tests (scavenging of superoxide, hydroxyl, and 1.1-diphenyl-2-picrylhydrazyl (DPPH) radical) were performed (Table 5). If 100% is observed as maximum antioxidant capacity, extracts of tested carrot organs had poor antioxidant activity; however, extracts of plants from 2017 had better antioxidant performance than that from 2016. In 2017, no changes due to infection were observed for any applied antioxidant test. Antioxidative capacity to scavenge superoxide radical did not change in 2016 as well, but the scavenging capacity of DPPH and hydroxyl radical increased more than twice in both plant organs. Interestingly, the induction of hydroxyl radical scavenging capacity increased 15 times. Antioxidant tests also revealed that infected leaves had better antioxidant capacity than roots, as an antioxidant response to oxidative stress induced by infection than root (Table 5).

## 3. Discussion

Stolbur Ca. Phytoplasma solani is highly distributed through different regions of Serbia since 1949, when it was discovered for the first time on pepper (*Capsicum annuum* L.) [22]. Ever since, disease symptoms caused by this pathogen have appeared on a wide range of diverse host plants, such as corn (*Zea mays* L.) [23], tobacco (*Nicotiana tabacum* L.) [24], parsnip (*Pastinaca sativa* L.) [25], grapevine (*Vitis vinifera* L.) [26], blueberry (*Vaccinium corymbosum* L.) [27], periwinkle (*Vinca minor* L.) [28], etc., depending on whether the transmission vector belongs to family Cixiidae or Cicadellidae [29]. Mixed infection, expressed through symptoms of leaves redness, shoot proliferation, and reduced tap roots quality, caused by phytoplasmas belonging to three 16S rRNA RFLP subgroups—Aster yellows group 16SrI (A and B) and Stolbur 16SrXII-A subgroup was found on carrot field in Serbia in 2007 [21]. This stolbur phytoplasma, transmitted by *Macrosteles laevis* (fam. Cicadellidae), was revealed to be one of the causal agents of carrot disease for the first time in Serbia. Mixed infection with both previously mentioned Ca. Phytoplasma solani groups (16SrI and 16SrXII) was also found in Slovenia, with specific distribution through the plant—in leaves (16SrI), between root and stem (16SrXII), and with both of them inhabiting root [30]. A 100% homology of Serbian Ca. Phytoplasma solani strains from carrot, with stolbur representatives from the NCBI database, indicates the infection caused by the phytoplasma belonging to group 16SrXII. As it is presented on the phylogenetic tree (Figure 3), sequencing of the 16S rRNA gene revealed low intra-species genetic diversity within Ca. Phytoplasma solani, regardless of the host or country of isolation. However, the detection of molecular variability within this species and other closely related species of genus *Candidatus* could be improved through the sequencing of some non-ribosomal DNA-fragments such as *tuf, secY*, or *vmp1* [31,32]. The previous phylogenetic study conducted by Quaglino et al. [33], with different Ca. Phytoplasma species (solani, australiense, japonicum, and fragariae), is in accordance with the results of phylogenetic analysis obtained in this study (Figure 3), probably indicating an environmentally influenced divergent evolution of these species from a common ancestor, resulting from different selection pressures and adaptation on new hosts. Carrot plants infected by Ca. Phytoplasma solani developed several of the characteristic symptoms for phytoplasmas [34], such as a growth arrest, a leaf reddening, and chlorophyll bleaching. A previous study reported that the leaf tissue of *O. biennis* plants was more vulnerable to the oxidative stress induced by Ca. Phytoplasma solani compared to the root [8]. Similarly, here, we present that leaves of carrots infected by Ca. Phytoplasma solani accumulated the product of oxidative degradation of membranes, MDA, to a much higher extent compared to the root, and with larger differences measured in 2017. In general, higher contents of soluble sugars and antioxidants, and lower levels of lipid peroxidation measured in both leaves and roots of asymptomatic plant branches for 2017 (Table 2), might be explained by the experience of drought stress due to much lower precipitation during June and by an induction of cross tolerance, thus by higher antioxidative capacity. In addition to polyphenols, which preferentially accumulated in symptomatic carrot plants in leaves, a significant role of GSH in the antioxidative defense of carrot leaves was indicated. However, a twice as large increase of reduced glutathione and total polyphenols (including anthocyanins) in symptomatic as in asymptomatic leaves of carrot were not efficient in the antioxidative protection against lipid peroxidation and degradation of photosynthetic pigments. Despite a much lower accumulation of total polyphenols and GSH (10%), the oxidative effect on lipids in roots was less pronounced (18%) compared to the leaf (61%). While roots of symptomatic plants accumulated carbohydrates by 25% and had a constitutively high amount of carotenoids, no changes were found in carbohydrate content in the infected leaves, as it has been reported for other species [8]. The activation of the phenylpropanoid pathway by a phytoplasma that results in differential accumulation of hydroxycinnamates, flavonoids, and anthocyanins has been shown for various species [7,8,35]. Results implied that tested leaves compared to the root of the carrot was preferentially exposed to the oxidative stress induced by Ca. Phytoplasma solani, which can be explained by (1) favoring the photosynthetic electron transfer to molecular oxygen that produces superoxide anion and other reactive oxygen species (ROS), and (2) impaired antioxidative action against superoxide anion of leaf extract. While root extracts also had poor scavenging capacity against superoxide, it is highly efficient against hydroxyl radical as it was shown by differential scavenging assays. Such activity was inducible, which means it was observed only in the roots of the symptomatic plants and might be ascribed to carbohydrates, which may be considered as a part of the plant antioxidative system [36]. 

## 4. Materials and Methods 

### 4.1. Symptoms Observation and Sampling of Plant Material

Carrot cultivar Bolero F-1 (Vilmorin, France) was planted in commercial fields in the second half of April covering 3 ha nearby Futog, Bačka region of Vojvodina, Northern Serbia (GPS: 45°15′14.04″ N, 19°39′35.01″ E in 2016; GPS: 45°15′11.71″ N, 19°39′24.65″ E in 2017). Monitoring and visual inspection of Ca. Phytoplasma solani development were performed bi-weekly during the carrot vegetation period. In the middle of July 2016 and 2017, for all experiments in the study, samples were collected from 20 points in the surveyed field, containing 40 symptomatic plants with intensely red leaves and 20 plants with no symptoms (Figure 1a,b). No symptoms of other plant pathogens were present on samples. One part of the plant material was lyophilized, and the other part (fresh) was immediately stored at temperature −80 °C. Extraction procedures were explained within each method. All experiments were performed in three replicates.

Meteorological data (temperature, precipitation) were monitored over the study years (2016–2017) from April to September in weather station Futog, nearest to the selected experimental field (45°14′ N, 19°42′ E) (Statistical Office of the Republic of Serbia). Averages monthly temperatures and precipitations were presented in Figure 2. 

### 4.2. Molecular Identification of Candidatus Phytoplasma Solani

A total DNA from 100 mg of freeze-dried symptomatic carrot leaves was extracted using a 2% CTAB extraction buffer, according to the protocol described by Li et al. [37]. Extracted DNA was re-suspended in 100 μL of TE buffer and stored at −70 °C until use. Nested PCR was performed to obtain nearly full-length 16S rDNA with an expected size of about 1.8 kb. The first round of PCR was performed with phytoplasma-specific universal primer pair P1/P7 (5′-AAGAGTTTGATCCTGGCTCAGGATT-3′/5′-CGTCCTTCATCGGCTCTT-3′) [38,39]. Diluted P1/P7 PCR products (1:10) were afterwards used as templates to perform another (nested) PCR amplification with P1A/P7A primer pair (5′-ACGCTGGCGGCGCGCCTAATAC-3′/5′-CCTTCATCGGCTCTTAGTGC-3′) [40]. PCR mix (20 μL) consisted of 12.4 µL of ultrapure DNase/RNase-free water, 2 µL of sample total DNA, 2 µL of 10 × KAPA Taq Buffer, 1.2 µL of 25 mM MgCl_2_, 0.2 µL of 20 mM dNTP mixture, 1 µL of each of the primer sets (10 µM), and 0.2 µL of KAPA Taq polymerase (5 U µL^−1^). PCR mix with 2 μL of DNAase/RNase-free water, instead of sample DNA, served as a negative control in each PCR reaction. Cycling conditions and the number of cycles for both primer pairs were set as follows: 2 min at 95 °C for initial denaturation, 34 cycles of denaturation for 60 s at 95 °C, annealing for 120 s at 50 °C, and extension for 180 s at 72 °C, followed by 10 min at 72 °C for final elongation step. PCR products were visualized on 1% agarose gel stained with ethidium bromide, and they were checked for a band presence on the predicted position in relation to a 1 kb GeneRuler DNA ladder (Sigma Life Science Online Product, Sigma-Aldrich, Germany, UK) under UV transilluminator. PCR products obtained with P1A/P7A primers were purified with Qiagen QIAquick PCR Purification Kit and sent for sequencing to Macrogen Europe B.V. Meibergdreef 31, 1105 AZ, Amsterdam, the Netherlands. The obtained sequence of one Serbian carrot strain generated in this study was deposited into the National Center for Biotechnology Information (NCBI) GenBank database to get an accession number. The sequence of Serbian carrot strain was used for further phylogenetic analysis and comparison with other sequences of Ca. Phytoplasma spp. available in the GenBank (http://www.ncbi.nlm.nih.gov/BLAST/ (accessed on 12 November 2020)). Phylogenetic analysis was performed to check the relatedness between strains of three formally described species within Stolbur phytoplasma group 16S rXII, the ones transmitted by polyphagous planthoppers from the Cixiidae family, to determine the position of tested Serbian strain in relation to them. For this purpose, sequences of Ca. Phytoplasma solani strains obtained from Serbia and other countries, affecting diverse host plants, as well as sequences of reference Ca. Phytoplasma strains (australiense, japonicum, and fragariae), retrieved from the NCBI database were used for phylogenetic analysis. Strains used for comparison are listed in Table 1. All sequences were aligned using ClustalW segment, implemented in BioEdit v. 7.0.5 program and used to construct a neighbor-joining phylogenetic tree in MEGA7 software. The bootstrap value for tree construction was set to 1000, and genetic distances were computed using the Kimura two-parameter nucleotide substitution model [41]. The tree was rooted with *Acholeplasma palmae* strain (Table 1).

### 4.3. Determination of Lipid Peroxidation Intensity (LP) and Total Carbohydrate Content (TCC)

The intensity of peroxidation of membrane lipids is measured by the amount of malondialdehyde (MDA), which is the secondary product of the oxidation of polyunsaturated fatty acids, as it is explained in method by Hodges et al. [42]. Thiobarbituric acid (TBA) forms a reaction medium featuring pink-red chromogen (maximal absorbance at 532 nm) with MDA and is measured against control without TBA. To exclude other interfering compounds (sugars, anthocyanins, and other phenolics), absorbance was measured at 440, 532 and 600 nm for correction. Lipid peroxidation intensity in carrot leaves and roots was calculated form formulas (1–3) and expressed as nmol MDA equivalents/g fresh weight (fw).

A = [(532–600)_abs+TBA_ − (532–600)_abs–TBA_] (1)

B = [(440–600)_abs+TBA_ − (440–600)_abs–TBA_] (2)

MDA equivalents (nmol/mL) = (A − B/157,000) × 10^6^ (3)

A modified sulfuric acid-UV method [43] was used for the determination of total carbohydrate content (TCC). One mL of aqueous plant extract (0.2 g/mL) was added to 3 mL of concentrated sulfuric acid, vortexed for 30 sec, and cooled in ice. After measuring the absorbance at 315 nm, calculation of TCC was performed using glucose as a standard. Results were expressed as mg glucose equivalents/g fw. Reduced glutathione (GSH) content was determined by Rahman et al. [44] and expressed as μmol reduced glutathione (GSH)/g fw.

### 4.4. Determination of PAL Activity, Total Polyphenols, Flavonoids, and Anthocyanins

Phenylalanine ammonia-lyase (PAL; EC 4.3.1.5) activity was performed according to the protocol given by Gerasimova et al. [45]. The content of cinnamic acid in the extracts was determined at 290 nm in reaction mixture containing *L*-phenylalanine against blank solution (1h at 37 °C in water-bath). The amount of cinnamic acid produced was determined from a trans-cinnamic acid standard curve, and PAL activity was expressed as U/g fw. 

Total polyphenols and tannins content was determined by Folin–Ciocalteu method [46] and expressed as gallic acid equivalents (GAE) in mg/g dry weight (dw). Reaction medium was 33% Folin–Ciocalteu phenol reagent, plant extract (50% MeOH), and 20% Na_2_CO_3_. Absorbance at 765 nm was recorded after 60 min of incubation at room temperature. The total tannin content was determined by the same Folin–Ciocalteu procedure after removal of tannins by adsorption on an insoluble matrix (polyvinylpolypyrrolidone, PVPP). Calculated values were subtracted from the total phenolic contents, and total tannin contents were expressed as mg GAE/g dw. The determination of total flavonoids content was performed according to Pękal and Pyrzynska [47] with plant extract (50% MeOH) and AlCl_3_ reagent (0.1 g of AlCl3 and 0.4 g of CH_3_COONa) in reaction medium. Absorbance was recorded at 430 nm against a blank, and the amount of flavonoids was calculated as a rutin equivalent from the calibration curve of rutin standard solutions and expressed as mg rutin/g dw. 

Proanthocyanidins were determined by a butanol-HCl assay [46]. Prepared MeOH extracts, butanol-HCl reagent (95:5 butanol-HCl), and 2% ferric reagent (2% ferric ammonium sulfate in 2.0 mol HCl), were kept in a boiling water-bath for 1 h. After cooling, absorbances were recorded at 550 nm against a blank without the extract. Proanthocyanidins were calculated as mg leucoanthocyanidin/g dw. Monomeric anthocyanins contents were determined using the differential method [48]. The absorbance of methanolic extracts with two buffer solutions at pH 1 and 4.5 was measured at 510 and 700 nm against a distilled water control. Total monomeric anthocyanin content was calculated as mg cyanidin-3-O-glucoside equivalents/g dw.

### 4.5. Determination of Chlorophylls and Carotenoids Contents

Chlorophyll a and b contents were determined according to method described by von Wettstein [49]. Fresh leaves were homogenized with 100% acetone by mortar with pestle and centrifuged (10 min at 4350× *g*). Absorbance was recorded at 440, 662, and 644 nm. Chlorophyll a and b contents were calculated from equations described in the applied method and given as mg/g dw. Total carotenoids content was determined according to a slightly modified method described by de Carvalho et al. [50]. Plant material, leaves, and roots were homogenized in a chilled mortar under the dim light and mixed with cold acetone and petroleum ether. Immediately afterwards, tubes were covered with aluminum foil, incubated (1 h in a cooled ultrasonic bath), and centrifuged (10 min at 12.857× *g*). Absorbance of the petroleum ether phase was recorded at 470 nm. The total carotenoid content was calculated from β-carotene standard curve and expressed as β-carotene equivalents in mg/g dw.

### 4.6. Determination of Antioxidant Capacity 

Antioxidant capacity was tested by three antioxidant tests that measure the scavenging activity of 1.1-diphenyl-2-picrylhydrazyl (DPPH) free radicals (DPPH test), superoxide anion (NBT test), and hydroxyl radical (•OH). The antioxidant activity of methanol dry extracts was assessed based on the DPPH test [51]. Another two tests were performed using fresh plant extracts, superoxide anion (O2•-) scavenging activity (NBT test) was based on a riboflavin-light-NBT system [52], and the hydroxyl radical (•OH) scavenging activity of extracts was assayed by the method of Sánchez-Moreno [53]. All scavenging activity tests were expressed as percentage (%) inhibition.

### 4.7. Data Analysis

Obtained results were expressed as means ± standard error and were tested using Student’s *t*-test (*p* < 0.05). Statistical analyses were performed using STATISTICA for Windows version 13 (Dell Inc., Aliso Viejo, CA, USA).

## 5. Conclusions

*Ca.* Phytoplasma solani accumulated the product of oxidative degradation of membranes, MDA, to much higher extent compared to the root. In addition to polyphenols, which preferentially accumulated in symptomatic carrot plants in leaves, a significant role of GSH in the antioxidative defense of carrot leaves was indicated. However, a twice as large increase of reduced glutathione and total polyphenols (including anthocyanins) in symptomatic as in asymptomatic leaves of carrot were not efficient in the antioxidative protection against lipid peroxidation and degradation of photosynthetic pigments. Despite a much smaller accumulation of total polyphenols and GSH (10%), the oxidative effect on lipids in roots was less pronounced (18%) compared to the leaves (61%). Results implied that leaves compared to the root of the carrot were preferentially exposed to the oxidative stress induced by Ca. Phytoplasma solani, which can be explained by (1) favoring the photosynthetic electron transfer to molecular oxygen that produces superoxide anion and other reactive oxygen species (ROS), and (2) impaired antioxidative action against the superoxide anion of leaves extracts.

## Figures and Tables

**Table 1:** Strains used for phylogenetic analysis.

Strain	Host	Country	Acc. Number
*Ca*. Phytoplasma solani			
121/09	Corn	Serbia	JQ730750
142/09	Tobacco	Serbia	JQ730739
224/09	Valerian	Serbia	JQ730742
231/09	Parsley	Serbia	JQ730741
138/10	Grapevine	Serbia	JQ730746
204/10	Periwinkle	Serbia	JQ730744
161/16	Parsnip	Serbia	KY579338
STOL11 (STOL) *	Periwinkle	France	AF248959
241/13	Corn	Bulgaria	KF907506
5043	Tomato	Greece	JX311953
G66	Pea	Poland	JN887313
SX-CP	Red sage	China	KT844645
06PS085	Grapevine	Canada	EU086529
Rus-PPT124 ^a^	Potato	Russia	EU344890
TPSP ^b^	Potato	Turkey	HM485579
BN-Op30 ^c^	Grapevine	Italy	EU836652
*Ca*. Phytoplasma australiense			
AGYP *	Grapevine	Australia	L76865
*Ca*. Phytoplasma japonicum *	Hydrangea	Japan	AB010425
*Ca*. Phytoplasma fragariae *	Strawberry	Lithuania	DQ086423
*Acholeplasma palmae*			L33734

* Reference strain for certain species. ^a^ Russia potato purple top phytoplasma (*Ca.* Phytoplasma solani strain causing potato purple top). ^b^ TPSP—Turkish potato stolbur phytoplasma (*Ca.* Phytoplasma solani strain causing potato stolbur). ^c^ Ca. Phytoplasma solani strain causing bois noir. AGYP—Australian grapevine yellows phytoplasma.

**Table 2:** Total carbohydrate content (%fresh weight (fw)), reduced glutathione content (GSH, μmol GSH/g fw), and lipid peroxidation intensity (nmol malondialdehyde (MDA)/g fw) in asymptomatic (A) and symptomatic (S) carrot leaves (L) and roots (R).

		2016			2017			
	Treatments	Mean	St. Error	A/S	Mean	St. Error	A/S	2016/2017
Total sugars	AL	5.79	0.01	*	6.99	0.52	*	*
	SL	5.62	0.00		16.61	0.59		*
	AR	4.10	0.01	*	12.94	1.48	*	*
	SR	5.15	0.01		16.03	0.75		*
Reduced glutathione	AL	2.75	0.01	*	6.94	0.05	*	*
	SL	5.49	0.01		17.88	0.11		*
	AR	3.10	0.01	*	2.56	0.05	*	*
	SR	3.29	0.01		8.92	0.19		*
Lipid peroxidation intensity	AL	871.57	0.26	*	70.00	3.70	*	*
	SL	1360.13	0.47		198.71	6.63		*
	AR	1064.43	3.80	*	108.84	6.17	*	*
	SR	1264.00	3.06		188.32	9.03		*

*—differ significantly at *p* < 0.05 (Student’s *t*-test).

**Table 3:** Phenylalanine ammonia-lyase (U/g fw) and polyphenolic compounds content (mg/g dw) in asymptomatic (A) and symptomatic (S) carrot leaves (L) and roots (R).

		2016			2017			
	Treatment	Mean	St. Error	A/S	Mean	St. Error	A/S	2016/2017
Phenylalanine ammonia-lyase activity	AL	258.82	0.29	nd	281.23	3.33	*	nd
	SL	336.71	11.00		518.32	12.42		*
	AR	70.94	0.03	nd	80.22	2.12	nd	nd
	SR	84.85	0.11		100.45	4.70		nd
Total polyphenols	AL	4.03	0.02	*	5.57	0.03	nd	nd
	SL	6.88	0.02		5.47	0.01		nd
	AR	1.42	0.01	*	1.13	0.01	nd	nd
	SR	1.92	0.01		1.34	0.00		nd
Total tannins	AL	3.33	0.04	*	2.32	0.28	nd	nd
	SL	3.94	0.01		2.70	0.09		nd
	AR	1.18	0.03	*	0.87	0.12	nd	nd
	SR	0.76	0.02		1.12	0.10		nd
Total flavonoids	AL	0.043	0.002	*	0.325	0.066	nd	*
	SL	0.100	0.006		0.235	0.078		*
	AR	0.000	0.000	nd	0.033	0.038	nd	*
	SR	0.000	0.000		0.025	0.075		*
Total proanthocyanidins	AL	0.40	0.00	*	0.003	0.00	*	*
	SL	0.15	0.00		0.001	0.00		*
	AR	6.02	0.00	*	0.001	0.00	*	*
	SR	4.87	0.01		0.002	0.00		*
Total anthocyanins	AL	0.003	0.00	*	0.088	0.00	*	*
	SL	0.798	0.00		0.025	0.00		*
	AR	0.00	0.00	*	0.00	0.00	nd	nd
	SR	0.00	0.00		0.00	0.00		nd

*—differ significantly at *p* < 0.05 (Student’s *t*-test), nd—no significant difference.

**Table 4:** Photosynthetic pigments (mg/g dw) in asymptomatic (A) and symptomatic (S) carrot leaves (L) and roots (R).

		2016			2017			
	Treatment	Mean	St. Error	A/S	Mean	St. Error	A/S	2016/2017
Total chlorophyll a	AL	0.78	0.01	*	0.25	0.08	*	*
	SL	0.24	0.01		0.10	0.02		*
	AR	0.13	0.01	nd	0.00	0.00	nd	nd
	SR	0.15	0.01		0.00	0.00		nd
Total chlorophyll b	AL	0.18	0.00	*	0.15	0.09	*	nd
	SL	0.15	0.00		0.06	0.04		*
	AR	0.25	0.00	nd	0.00	0.00	nd	*
	SR	0.28	0.01		0.00	0.00		*
Total carotenoids	AL	0.36	0.01	*	0.009	0.02	*	*
	SL	0.17	0.00		0.002	0.02		*
	AR	1.94	0.00	nd	0.200	0.02	nd	nd
	SR	1.59	0.00		0.170	0.05		*

*—differ significantly at *p* < 0.05 (Student’s *t*-test), nd—no significant difference.

**Table 5:** Antioxidant capacity (% of neutralized radicals) in asymptomatic (A) and symptomatic (S) carrot leaves (L) and roots (R).

		2016			2017			
Antioxidant test	Treatment	Mean	St. Error	A/S	Mean	St. Error	A/S	2016/2017
NBT-test	AL	20.33	0.28	nd	55.89	0.10	nd	*
	SL	19.80	0.06		54.93	0.02		*
	AR	82.57	0.23	*	67.94	0.02	nd	*
	SR	99.23	0.09		80.95	0.00		nd
^•^OH-test	AL	10.81	0.69	*	69.68	0.06	nd	*
	SL	20.32	1.93		55.98	0.01		*
	AR	2.42	0.04	*	11.54	0.01	nd	*
	AL	39.97	4.16		13.75	0.00		*
DPPH-test	SL	19.95	0.02	*	55.99	0.15	nd	*
	AR	32.58	0.22		48.09	1.17		*
	SR	10.90	0.01	*	19.52	1.00	nd	*
	SR	21.25	0.01		18.49	1.47		nd

*—differ significantly at *p* < 0.05 (Student’s *t*-test), nd—no significant difference.

## References

[1] Lee I.M., Davis R.E., Gundersen-Rindal D.E. (2000). Phytoplasma: Phytopathogenic mollicutes. Annu. Rev. Microbiol..

[2] Ermacora P., Osler R., Musetti R., Pagliari L. (2019). Symptoms of Phytoplasma Diseases. Phytoplasmas. Methods in Molecular Biology.

[3] Kumari S., Krishnan N., Rai A.B., Singh B., Rao G.P., Bertaccini A. (2019). Global status of phytoplasma diseases in vegetable crops. Front. Microbiol..

[4] Foyer C.H., Noctor G. (2013). Redox Signaling in Plants. Antioxid. Redox Signal.

[5] Kazan K., Lyons R. (2014). Intervention of phytohormone pathways by pathogen effectors. Plant Cell.

[6] Tomkins M., Kliot A., Marée A.F., Hogenhout S.A. (2018). A multi-layered mechanistic modelling approach to understand how effector genes extend beyond phytoplasma to modulate plant hosts, insect vectors and the environment. Curr. Opin. Plant Biol..

[7] Musetti R., Weintraub P.G., Jones P. (2010). Biochemical changes in plants infected by phytoplasmas. Phytoplasmas: Genomes, Plant Hosts and Vectors.

[8] Kiprovski B., Đalović I., Adamović D., Mitrović P., Marjanović-Jeromela A., Malenčić Đ., Popović T. (2018). Biochemical changes in *Oenothera biennis* plants infected by ‘*Candidatus* Phytoplasma solani’. J. Plant Pathol..

[9] Malamy J., Sanchez-Casas P., Hennig J., Guo A., Klessig D.F. (1996). Dissection of the salicylic acid signaling pathway in tobacco. Mol. Plant Microbe Interact. MPMI.

[10] Mur L.A.J., Bi Y.M., Darby R.M., Firek S., Draper J. (1997). Compromising early salicylic acid accumulation delays the hypersensitive response and increases viral dispersal during lesion establishment in TMV-infected tobacco. Plant J..

[11] Shirasu K., Nakajima H., Rajasekhar V.K., Dixon R.A., Lamb C. (1997). Salicylic acid potentiates an agonist-dependent gain control that amplifies pathogen signals in the activation of defense mechanisms. Plant Cell.

[12] Dempsey D.M.A., Shah J., Klessig D.F. (1999). Salicylic acid and disease resistance in plants. Crit. Rev. Plant Sci..

[13] Felton G.W., Korth K.L., Bi J.L., Wesley S.V., Huhman D.V., Mathews M.C., Murphy J.B., Lamb C., Dixon R.A. (1999). Inverse relationship between systemic resistance of plants to microorganisms and to insect herbivory. Curr. Biol..

[14] Rice-Evans C.A., Miller N.J., Bolwell P.G., Bramley P.M., Pridham J.B. (1995). The relative antioxidant activities of plant-derived polyphenolic flavonoids. Free Radic. Res..

[15] Williams R.J., Spencer J.P., Rice-Evans C. (2004). Flavonoids: Antioxidants or signalling molecules?. Free Radic. Biol. Med..

[16] Jović J., Cvrković T., Mitrović M., Krnjajić S., Petrović A., Redinbaugh G.M., Pratt C.R., Hogenhout A.S., Toševski I. (2009). Stolbur Phytoplasma transmission to maize by Reptalus panzeri and the disease cycle of maize redness in Serbia. Phytopathology.

[17] Adamović D., Đalović I., Mitrović P., Kojić S., Starović M., Purar B., Jošić D. (2014). First report of 16SrXII-A subgroup Phytoplasma (Stolbur) associated with reddening of *Oenothera biennis* in Serbia. Plant Dis..

[18] Starović M., Pavlović S., Stojanović S., Jošić D. (2015). Medicinal plants phytoplasma. Plant Prot..

[19] Mitrović P., Trkulja V., Adamović D., Đalović I., Milovac Ž., Kovačić–Jošić D., Mihić–Salapura J. (2016). First report of Stolbur Phytoplasma on Mentha x piperita in Serbia. Plant Dis..

[20] Duduk B., Bulajić A., Duduk N., Calari A., Paltrinieri S., Krstić B., Bertaccini A. (2007). Identification of phytoplasmas belonging to aster yellows ribosomal group in vegetables in Serbia. Bull. Insectology.

[21] Duduk B., Perić P., Marčić D., Drobnjaković T., Picciau L., Alma A., Bertaccini A. (2008). Phytoplasmas in carrots disease and potential vectors in Serbia. Bull. Insectol..

[22] Martinović M., Bjegović P. (1950). O nekim bolestima i štetočinama utvrđenim u NR Srbiji u 1949 godini. Plant Protect..

[23] Duduk B., Bertaccini A. (2006). Corn with symptoms of reddening: New host of stolbur phytoplasma. Plant Dis..

[24] Mitrović J., Duduk B. (2011). Occurrence of a new stolbur strain in tobacco in Serbia. Bull. Insectol..

[25] Medić Pap S., Gvozdanović Varga J., Červenski J., Stepanović J., Rekanović E., Stepanović M., Duduk B. (2018). First Report of ‘*Candidatus* Phytoplasma solani’Infecting Parsnip in Serbia. Plant Dis..

[26] Duduk B., Botti S., Ivanović M., Krstić B., Dukić N., Bertaccini A. (2004). Identification of phytoplasmas associated with grapevine yellows in Serbia. J. Phytopathol..

[27] Starović M., Kojić S., Kuzmanović S.T., Stojanović S.D., Pavlović S., Jošić D. (2013). First Report of Blueberry Reddening Disease in Serbia Associated with 16SrXIIA (Stolbur) Phytoplasma. Plant Dis..

[28] Mitrović J., Pavlović S., Duduk B. (2013). Survey and multigene characterization of stolbur phytoplasmas on various plant species in Serbia. Phytopathol. Mediterr..

[29] Maixner M. (2011). Recent advances in Bois noir research. Petria.

[30] Mehle N., Mermal S., Vidmar S., Marn M.V., Dreo T., Dermastia M. (2018). First Report of Carrot Infection with Phytoplasmas in Slovenia. V: Bois Noir 5th Workshop.

[31] Langer M., Maixner M. (2004). Molecular characterisation of grapevine yellows associated phytoplasmas of the stolbur-group based on RFLP–analysis of non-ribosomal DNA. Vitis.

[32] Cimerman A., Pacifico D., Salar P., Marzachì C., Foissac X. (2009). Striking diversity of vmp1, a variable gene encoding a putative membrane protein of the stolbur phytoplasma. Appl. Environ. Microbiol..

[33] Quaglino F., Zhao Y., Casati P., Bulgari D., Bianco P.A., Wei W., Davis R.E. (2013). ‘Candidatus Phytoplasma solani’, a novel taxon associated with stolbur-and bois noir-related diseases of plants. Int. J. Syst. Evol. Microbiol..

[34] Hogenhout S.A., Oshima K., Ammar E.D., Kakizawa S., Kingdom H.N., Namba S. (2008). Phytoplasmas: Bacteria that manipulate plants and insects. Mol. Plant Pathol..

[35] Himeno M., Kitazawa Y., Yoshida T., Maejima K., Yamaji Y., Oshima K., Namba S. (2014). Purple top symptoms are associated with reduction of leaf cell death in phytoplasma-infected plants. Sci. Rep..

[36] Matros A., Peshev D., Peukert M., Mock H.P., van den Ende W. (2015). Sugars as hydroxyl radical scavengers: Proof-of-concept by studying the fate of sucralose in *Arabidopsis*.. Plant J..

[37] Li R., Mock R., Huang Q., Abad J., Hartung J., Kinard G. (2008). A reliable and inexpensive method of nucleic acid extraction for the PCR-based detection of diverse plant pathogens. J. Virol. Methods.

[38] Deng S., Hiruki C. (1991). Amplification of 16S rRNA genes from culturable and nonculturable mollicutes. J. Microbiol. Methods..

[39] Schneider B., Seemuller E., Smart C.D., Kirkpatrick B.C., Razin S., Tully J.G. (1995). Phylogenetic classification of plant pathogenetic mycoplasmalike organisms or phytoplasmas. Molecular and Diagnostic Procedures in Mycoplasmology.

[40] Lee M., Martini M., Marcone C., Zhu S.F. (2004). Classification of phytoplasma strains in the elm yellows group (16SrV) and proposal of ‘Candidatus Phytoplasma ulmi’ for the phytoplasma associated with elm yellows. Int. J. Syst. Evol. Microbiol..

[41] Kimura M. (1980). A simple method for estimating evolutionary rates of base substitutions through comparative studies of nucleotide sequences. J. Mol. Evol..

[42] Hodges D.M., DeLong J.M., Forney C.F., Prange R.K. (1999). Improving the thiobarbituric acid-reactive-substances assay for estimating lipid peroxidation in plant tissues containing anthocyanin and other interfering compounds. Planta.

[43] Albalasmeh A.A., Berhe A.A., Ghezzehei T.A. (2013). A new method for rapid determination of carbohydrate and total carbon concentrations using UV spectrophotometry. Carbohydr. Polym..

[44] Rahman I., Kode A., Biswas S.K. (2006). Assay for quantitative determination of glutathione and glutathione disulfide levels using enzymatic recycling method. Nat. Protoc..

[45] Gerasimova N.G., Pridvorova S.M., Ozeretskovskaya O.L. (2005). Role of L–phenylalanine ammonia Lyase in the induced resistance and susceptibility of sotato plants. Appl. Biochem. Microbiol..

[46] Makkar H.P.S. (2003). Quantification of Tannins in Tree and Shrub Foliage: A Laboratory Manual.

[47] Pękal A., Pyrzynska K. (2014). Evaluation of aluminium complexation reaction for flavonoid content assay. Food Anal. Methods.

[48] Lee J., Durst R.W., Wrolstad R.E. (2005). Determination of total monomeric anthocyanin pigment content of fruit juices, beverages, natural colorants and wines by the pH differential method: Collaborative study. J. AOAC Int..

[49] Von Wettstein D. (1957). Chlorophyll-letale und der submikroskopische Formwechsel der plastiden. Exp. Cell Res..

[50] De Carvalho L.M.J., Gomes P.B., de Oliveira Godoy R.L., Pacheco S., do Monte P.H.F., de Carvalho J.L.V., Nutti M.R., Neves A.C.L., Vieira A., Ramos S.R.R. (2012). Total carotenoid content, α-carotene and β-carotene, of landrace pumpkins (*Cucurbita moschata* Duch): A preliminary study. Food Res. Int..

[51] Panda S.K., El-Missiry M.A. (2012). Assay guided comparison for enzymatic andnonenzymatic antioxidant activities with special reference to medicinal plants. Antioxidant Enzyme.

[52] Ahmed D., Saman Z., Hira B. (2013). In vitro analysis of antioxidant activities of *Oxalis corniculata* Linn. fractions in various solvents. Afr. J. Tradit. Complement Altern. Med..

[53] Sánchez-Moreno C. (2002). Methods used to evaluate the free radical scavenging activity in foods and biological systems. Int. J. Food Sci. Technol..

